# Sitagliptin and heart failure hospitalization in patients with type 2 diabetes

**DOI:** 10.18632/oncotarget.10507

**Published:** 2016-07-09

**Authors:** Chin-Hsiao Tseng

**Affiliations:** ^1^ Department of Internal Medicine, National Taiwan University College of Medicine, Taipei, Taiwan; ^2^ Division of Endocrinology and Metabolism, Department of Internal Medicine, National Taiwan University Hospital, Taipei, Taiwan; ^3^ Division of Environmental Health and Occupational Medicine of the National Health Research Institutes, Zhunan, Taiwan

**Keywords:** heart failure, hospitalization, incretin, sitagliptin, Taiwan

## Abstract

This study evaluated the risk of heart failure hospitalization in a 1:1 matched pair sample of sitagliptin ever and never users derived from the Taiwan's National Health Insurance. A total of 85,859 ever users and 85,859 never users matched on 8 digits of propensity score were followed for the first event of heart failure hospitalization until December 31, 2011. The treatment effect (forever versus never users, and for tertiles of cumulative duration of therapy) was estimated by Cox regression incorporated with the inverse probability of treatment weighting using propensity score. Additionally, adjusted hazard ratios for heart failure were estimated for the baseline characteristics in sitagliptin ever users. Results showed that the incidence of heart failure hospitalization was 1,020.16 and 832.54 per 100,000 person-years, respectively, for ever and never users, with an overall hazard ratio (95% confidence intervals) of 1.262 (1.167-1.364). While compared to never users, the respective hazard ratio for the first, second, and third tertile of cumulative duration < 3.7, 3.7-10.3 and >10.3 months was 2.721 (2.449-3.023), 1.472 (1.318-1.645) and 0.515 (0.447-0.594). Older age, longer diabetes duration, male sex, and use of insulin, sulfonylurea, calcium channel blockers, aspirin, ticlopidine, clopidogrel and dipyridamole were significantly associated with a higher risk in sitagliptin users, but dyslipidemia and use of metformin and statin were protective. In conclusion, sitagliptin increases the risk of heart failure hospitalization within one year of its use, but reduces the risk thereafter. Some factors predisposing to sitagliptin-related heart failure are worthy of attention in clinical practice.

## INTRODUCTION

Two clinical trials published in 2013 brought to public concern a possible risk of heart failure associated with the use of dipeptidyl-peptidase 4 (DPP-4) inhibitors in the treatment of type 2 diabetes mellitus. The Saxagliptin Assessment of Vascular Outcomes Recorded in Patients with Diabetes Mellitus (SAVOR)-Thrombolysis in Myocardial Infarction (TIMI) 53 trial (SAVOR-TIMI53) showed an unexpectedly higher risk of heart failure hospitalization in patients treated with saxagliptin *vs*. placebo [3.5% *vs*. 2.8%; hazard ratio 1.27, 95% confidence interval (CI): 1.07-1.51, *P* = 0.007] [1]. Although not significant, more patients treated with alogliptin were diagnosed with heart failure than patients taking placebo, as demonstrated in the Examination of Cardiovascular Outcomes with Alogliptin *versus* Standard of Care (EXAMINE) [2, 3]. In the meta-analysis by Monami et al. when these two clinical trials were pooled together, the estimated Mantel-Haenszel odds ratio was 1.24 (95% CI: 1.07-1.45, *P* = 0.004) [3]. However, such an increased risk of heart failure was not similarly observed in the more recently published Trial Evaluating Cardiovascular Outcomes with Sitagliptin (TECOS), which suggested a neutral risk association between sitagliptin use and placebo, with an estimated hazard ratio of 1.00 (95% CI: 0.83-1.20, *P* = 0.98) [4].

Four independent meta-analyses published in 2014 did not make a consistent conclusion. Iqbal et al. estimated a pooled incidence rate ratio (95% CI) of 0.55 (0.27-1.12) for heart failure associated with saxagliptin from 20 clinical trials [5]. Monami et al. estimated a Mantel-Haenszel odds ratio of 1.19 (95% CI: 1.03-1.37, *P* = 0.015) for DPP-4 inhbitors from 84 randomized trials up to October 1, 2013 [3]. When different DPP-4 inhibitors were estimated separately, the Mantel-Haenszel odds ratio (95% CI) was 0.99 (0.44-2.24), 0.55 (0.20-1.53), 1.22 (1.03-1.45), 1.56 (0.66-3.65) and 1.18 (0.89-1.56), respectively, for sitagliptin, vildagliptin, saxagliptin, linagliptin and alogliptin [3]. Savarese et al. included 94 randomized trials in their meta-analysis and found that long-term (29 weeks or more) use of DPP-4 inhibitors (not specified) was associated with a significantly higher risk of heart failure (relative risk 1.158, 95% CI: 1.011-1.326, *P* = 0.034), but this was not observed in short-term users (relative risk 0.668, 95% CI: 0.318-1.400, *P* = 0.285) [6].

In the fourth meta-analysis, Clifton included 4 cohort studies and 5 randomized trials (including SAVOR-TIMI53 and EXAMINE) published since October 2013 and estimated an odds ratio of 1.148 (95% CI: 1.025-1.287, *P* = 0.017) for DPP-4 inhibitors [7]. When cohort studies and clinical trials were analyzed separately, only the odds ratio derived from the 5 clinical trials was significant (1.239, 95% CI: 1.078-1.424, *P* = 0.002), and that derived from the 4 cohort studies was not (1.099, 95% CI: 0.913-1.323, *P* = 0.317) [7].

It is worthy to note that the studies included in the fourth meta-analysis were restricted to recent publications and only one cohort study by Weir et al. was focused on the effect of sitagliptin by using a nested case-control design to analyze the US claims database from a nationally based commercial insurance [8]. They showed that sitagliptin increased the risk of heart failure hospitalization among diabetic patients with pre-existing heart failure (12.5% *vs*. 9.0%), with an adjusted odds ratio of 1.84 (95% CI: 1.16-2.92) [8].

Sitagliptin was the first DPP4 inhibitor approved in Taiwan on July 13, 2007, while the other DPP4 inhibitors (i.e., saxagliptin, vildagliptin and linagliptin) were not approved until after 2009 [9]. A recent study by Wang et al. used a 1:1 matched pairs of users and non-users of sitagliptin in the reimbursement database of the National Health Insurance (NHI) in Taiwan [10]. They showed that the adjusted hazard ratio for the first event of heart failure hospitalization for sitagliptin was 1.21 (95% CI 1.04-1.42, *P* = 0.017) [10].

Therefore, whether the most commonly used DPP-4 inhibitor, sitagliptin, may increase the risk of heart failure is under-investigated and inconclusive. While the meta-analysis by Monami et al. [3] including 11 randomized trials suggested a null association, the two analyses of insurance databases showed a significantly higher risk [8, 10]. Because not many of these studies evaluated heart failure risk with regards to exposure duration, the present study aimed at evaluating whether sitagliptin use would affect the risk differently among different groups of exposure duration by using the reimbursement database of the NHI. Other incretins were not evaluated because they were not used commonly during the study period. In addition, a new-user design was used to minimize the potential “prevalent user bias” [11]. To reduce the potential risk of “immortal time bias” (the initial period of follow-up during which the outcome can not occur) [12, 13], patients included into the study should have been prescribed antidiabetic drugs for at least two times. To avoid the potential confounding from the differences in baseline characteristics associated with treatment allocation in non-random observational studies, a 1:1 matched-pair sample based on 8 digits of propensity score (PS) was used according to the methods described by Parsons [14]. Calculation of standardized difference for each baseline characteristic as recommended by Austin and Stuart [15] was used as a formal test for balance diagnostics. To obtained unbiased estimates, Cox regression models were created by incorporation with the inverse probability of treatment weighting (IPTW) using PS as recommended by Austin [16].

## RESULTS

Table 1 compares the baseline characteristics between ever users and never users of sitagliptin in the matched-pair sample. Although 15 among the 30 variables were significantly different between ever and never users with *P* < 0.05, none of the variables had a standardized difference > 10%, suggesting that residual confounding from the baseline characteristics might not be remarkable [15].

**Table 1:** Baseline characteristics of sitagliptin never users and ever users

Variables	Sitagliptin				*P*	Standardized difference
	Never users (*n* = 85859)		Ever users (*n* = 85859)			
	*n*	%	*n*	%		
Age (years)*	54.90 ± 13.30		54.96 ± 12.15		0.3214	0.43
Diabetes duration (years)*	3.20 ± 2.64		3.27 ± 2.69		<0.0001	2.81
Sex (men)	47171	54.94	46687	54.38	0.0190	−1.22
Hypertension	54094	63.00	54568	63.56	0.0176	1.02
Chronic obstructive pulmonary disease	29728	34.62	30187	35.16	0.0201	1.18
Stroke	14109	16.43	14271	16.62	0.2926	0.50
Nephropathy	11901	13.86	12067	14.05	0.2477	0.60
Ischemic heart disease	25359	29.54	25784	30.03	0.0249	1.09
Peripheral arterial disease	10892	12.69	11031	12.85	0.3148	0.49
Obesity	4434	5.16	4531	5.28	0.2927	0.48
Dyslipidemia	53409	62.21	53775	62.63	0.0682	0.85
Acute pancreatitis	2248	2.62	2205	2.57	0.5138	−0.32
Alcohol-related diagnoses	3939	4.59	3878	4.52	0.4801	−0.38
Cancer	54	0.06	65	0.08	0.3131	0.47
Sulfonylurea	60850	70.87	60309	70.24	0.0042	−1.67
Metformin	60705	70.70	60099	70.00	0.0014	−1.82
Meglitinides	10284	11.98	10327	12.03	0.7495	0.11
Acarbose	13887	16.17	14292	16.65	0.0083	1.15
Pioglitazone	5917	6.89	6599	7.69	<0.0001	2.93
Rosiglitazone	11975	13.95	12513	14.57	0.0002	1.70
Insulin	9320	10.86	9023	10.51	0.0203	−1.01
Statin	34256	39.90	34671	40.38	0.0410	0.90
Fibrate	23894	27.83	24267	28.26	0.0451	0.91
Angiotensin converting enzyme inhibitor	34804	40.54	35156	40.95	0.0838	0.75
Angiotensin receptor blocker	27222	31.71	27685	32.24	0.0166	1.08
Calcium channel blocker	37362	43.52	37589	43.78	0.2694	0.47
Aspirin	35529	41.38	35942	41.86	0.0432	0.95
Ticlopidine	2079	2.42	2158	2.51	0.2191	0.60
Clopidogrel	3336	3.89	3327	3.87	0.9105	−0.04
Dipyridamole	22728	26.47	22943	26.72	0.2403	0.56

* Age and diabetes duration are compared by Student's t test and expressed as mean and standard deviation, other variables are compared by Chi square test.

Table 2 shows the incidence of heart failure hospitalization by sitagliptin exposure and the hazard ratios comparing sitagliptin exposed to unexposed. During follow-up, a total of 2,988 never users and 1,134 ever users developed first events of heart failure hospitalization, with respective incidence of 832.54 and 1,020.16 per 100,000 person-years. The overall hazard ratio was 1.262 (1.167-1.364). For the tertiles of cumulative duration of sitagliptin therapy, significantly increased risk was observed for the first and second tertiles, but the risk was significantly reduced in the third tertiles.

**Table 2:** Incidence of heart failure hospitalization by sitagliptin exposure and the hazard ratios comparing sitagliptin exposed to unexposed

Sitagliptin use	Case number followed	Incident cases of heart failure hospitalization	Person-years	Incidence rate of heart failure hospitalization (per 100,000 person-years)	Hazard ratio (95% confidence interval)	*P*
Never users	85859	2988	358903.58	832.54	1.000	
Ever users	85859	1134	111158.84	1020.16	1.262 (1.167-1.364)	<0.0001
**Cumulative duration (months)**						
Never users	85859	2988	358903.58	832.54	1.000	
<3.7	26643	498	20635.73	2413.29	2.721 (2.449-3.023)	<0.0001
3.7-10.3	30491	418	35868.65	1165.36	1.472 (1.318-1.645)	<0.0001
>10.3	28725	218	54654.47	398.87	0.515 (0.447-0.594)	<0.0001

Table 3 shows the adjusted hazard ratios for heart failure hospitalization for all baseline characteristics in patients ever treated with sitagliptin. Older age, longer diabetes duration, male sex, and use of insulin, sulfonylurea, calcium channel blockers, aspirin, ticlopidine, clopidogrel and dipyridamole were significantly associated with a higher risk, but dyslipidemia and use of metformin and statin were associated with a significantly lower risk.

**Table 3:** Adjusted hazard ratios for heart failure hospitalization in patients ever treated with sitagliptin

Variable	Interpretation	Hazard ratio (95% confidence interval)	*P*
Age	Every 1-year increment	1.051 (1.045-1.057)	<0.0001
Diabetes duration	Every 1-year increment	1.171 (1.039-1.320)	0.0098
Sex	Men *versus* women	1.033 (1.007-1.061)	0.0145
Hypertension	Yes *versus* no	0.918 (0.761-1.107)	0.3713
Chronic obstructive pulmonary disease	Yes *versus* no	1.028 (0.910-1.162)	0.6534
Stroke	Yes *versus* no	1.009 (0.875-1.164)	0.9049
Nephropathy	Yes *versus* no	1.276 (1.105-1.472)	0.9049
Ischemic heart disease	Yes *versus* no	1.093 (0.952-1.255)	0.2082
Peripheral arterial disease	Yes *versus* no	1.077 (0.926-1.252)	0.3348
Obesity	Yes *versus* no	1.103 (0.820-1.483)	0.5174
Dyslipidemia	Yes *versus* no	0.768 (0.666-0.885)	0.0003
Acute pancreatitis	Yes *versus* no	1.214 (0.852-1.729)	0.2827
Alcohol-related diagnoses	Yes *versus* no	1.325 (1.010-1.739)	0.0419
Cancer	Yes *versus* no	2.831 (0.703-11.405)	0.1433
Sulfonylurea	Yes *versus* no	1.286 (1.085-1.526)	0.0038
Metformin	Yes *versus* no	0.791 (0.678-0.923)	0.0030
Meglitinides	Yes *versus* no	1.126 (0.958-1.323)	0.1489
Acarbose	Yes *versus* no	1.049 (0.901-1.220)	0.5386
Pioglitazone	Yes *versus* no	1.021 (0.832-1.254)	0.8407
Rosiglitazone	Yes *versus* no	0.983 (0.834-1.158)	0.8386
Insulin	Yes *versus* no	1.483 (1.264-1.739)	<0.0001
Statin	Yes *versus* no	0.831 (0.723-0.955)	0.0089
Fibrate	Yes *versus* no	0.963 (0.837-1.108)	0.6009
Angiotensin converting enzyme inhibitor	Yes *versus* no	1.115 (0.964-1.289)	0.1442
Angiotensin receptor blocker	Yes *versus* no	1.129 (0.986-1.293)	0.0783
Calcium channel blocker	Yes *versus* no	1.193 (1.020-1.394)	0.0269
Aspirin	Yes *versus* no	1.199 (1.040-1.383)	0.0125
Ticlopidine	Yes *versus* no	1.256 (0.981-1.608)	0.0707
Clopidogrel	Yes *versus* no	1.468 (1.190-1.812)	0.0003
Dipyridamole	Yes *versus* no	1.333 (1.166-1.525)	<0.0001

## DISCUSSION

The present study confirmed the findings of an overall increased risk of heart failure hospitalization associated with sitagliptin use as observed in a previous analysis that also used the reimbursement database of the NHI [10]. The estimated overall hazard ratio of 1.262 (95% CI 1.167-1.364) in the present study (Table 2) was very close to the estimated 1.21 (95% CI 1.04-1.42) observed in the previous study [10]. Besides, the present study suggested that such an increased risk was mainly observed during a short duration of its use, i.e., with a cumulative duration of therapy < 1 year (Table 2). After long-term use, the risk was actually significantly reduced (Table 2). The present study also identified some predictive and some protective factors in association with heart failure hospitalization in sitagliptin users (Table 3). The predictive factors included an older age, a longer diabetes duration, male sex and use of insulin, sulfonylurea, calcium channel blockers and anti-platelet drugs (Table 3). On the other hand, the protective factors included dyslipidemia and use of metformin and statin (Table 3).

A significantly higher risk of heart failure hospitalization associated with short-term rather than long-term use of sitagliptin (Table 2) was contradictory to the finding in the meta-analysis by Savarese et al. [6], which showed a significantly higher risk associated with long-term use of DPP-4 inhibitors for 29 weeks or more. Because this meta-analysis did not consider separate DPP-4 inhibitors, it is not known whether the discrepant findings could be due to the undifferentiation of the DPP-4 inhibitors in the meta-analysis.

The lower risk associated with sitagliptin after a longer duration of its exposure (Table 2) suggested a potentially protective effect of sitagliptin on heart failure. Such a protective effect could be supported by a recent study by dos Santos et al. conducted in human and experimental heart failure [17]. The investigators showed an increase of approximately 130% in circulating DPP-4 activity in patients with heart failure and an inverse correlation between serum DPP-4 activity and left ventricular ejection fraction in patients with heart failure [17]. Furthermore, long-term sitagliptin treatment for 6 weeks significantly improved cardiac performance and mitigated the development and progression of heart failure in rats [17]. Such a beneficial effect of sitagliptin on heart failure could also be demonstrated in another study using pigs as a model [18], but could not be similarly shown when vildagliptin was used in rats [19], suggesting that the protective effect of sitagliptin on heart failure might not be a class effect of DPP-4 inhibitors. However, the observed “protective effect” of sitagliptin after its long-term use may also be explained by reasons not directly related to a real protective effect of the drug on heart failure. First, the reduced risk of heart failure after long-term use might be resulted from the better long-term diabetes control in the users. Second, the lower risk after long-term sitagliptin therapy could be explained by a depletion of susceptible cases. Patients who were predisposed to heart failure might have exhibited the event soon after their use of sitagliptin, leaving the remaining in the cohort of long-term therapy less susceptible to heart failure.

On the other hand, the significantly higher risk of heart failure hospitalization within a short-term exposure to sitagliptin might either be due to the drug per se or due to some other reasons not necessarily indicating a cause-effect relationship. First, the care-givers would be more cautious in the search of severe adverse events when they prescribed a new drug to their patients, leading to a possible detection bias among new users. However, possible detection bias due to the awareness and alertness of heart failure related to the use of DPP-4 inhibitors was less likely because the two clinical trials (i.e., SAVOR-TIMI53 and EXAMINE) reporting an unexpectedly higher risk of heart failure hospitalization related to the use of saxagliptin and alogliptin were published in 2013 [1, 2] and the follow-up of the present study cohort was actually ended before this year. Second, although all standardized differences were < 10% and did not suggest a residual confounding from the differences in the baseline characteristics, 15 out of the 30 variables were significantly different between ever and never users of sitagliptin (Table 1). It is worthy to point out that many of the ever users of sitagliptin were characterized by significantly higher prevalence of risk factors of heart failure such as ischemic heart disease, chronic obstructive pulmonary disease, hypertension and use of thiazolidinediones (Table 1). Therefore, it is not known whether residual confounding from these potential risk factors or from some unmeasured risk factors could exert an effect on the observation of a significantly higher risk of heart failure in short-term users of sitagliptin (Table 2).

The discrepant risk association with regards to exposure duration is an interesting finding that has not been reported previously. Estimating an overall risk by lumping together all drug users into an exposure group without considering different durations of exposure would not provide full information and might not be adequate in data analyses. Studies mainly including patients within the first year of sitagliptin use may overestimate an overall risk, but studies including more patients with longer duration of exposure might find an attenuated relative risk and concluded with a neutral or even a protective effect.

The discrepant findings between the present study and the recently published TECOS clinical trial which showed a lack of increased risk of heart failure in the sitagliptin group [4] are worthy of discussion. First, the TECOS study is a clinical trial and the generalization of the findings should be limited to patients fitting the inclusion and exclusion criteria set in the trial. On the other hand, the present study mainly reflected a real world scenario. For example, in the TECOS, patients were recruited with a glycated hemoglobin level of 6.5% to 8.0% at enrollment [4]. However, in clinical practice in Taiwan, sitagliptin is mainly used as a second or third line therapy because of its higher cost in relative to other preexisting oral antidiabetic drugs. As a result, most patients would be prescribed sitagliptin only when other treatment modalities could not adequately control the blood glucose level, say with a glycated hemoglobin level of > 8.0%. Therefore, the observed outcomes derived from a clinical trial may not be necessarily the same when a medication is widely used in clinical practice in the real world. This discrepancy can also be supported by other observational studies previously conducted in Taiwan [10] and in the USA [8], both reporting a significantly higher risk of heart failure associated with sitagliptin use. Second, ethnicity difference may also partly explain the discrepant findings between the TECOS and the present study. While the present study recruited a more homogeneous group of patients, the TECOS trial was conducted mainly in a mixture of different ethnicities (67.9% white, 22.3% Asian, 3.0% black and 6.8% others). It has already been known that DPP-4 inhibitors may have a better efficacy on blood glucose lowering in the Asian populations than in the western people [20], suggesting that the adverse effects related to incretin-based therapy may also show ethnicity difference.

The identification of predictive and protective factors associated with heart failure hospitalization in sitgaliptin users may have some clinical implications. The predictive factors suggested that sitagliptin use should be closely observed for the potential risk of heart failure in patients with an older age, a longer diabetes duration, male sex and use of insulin, sulfonylurea, calcium channel blockers and anti-platelet drugs (Table 3). On the other hand, dyslipidemia and use of metformin and statin might show a protective effect (Table 3). It is not known whether the lower risk of heart failure associated with dyslipidemia could be due to the protective effect of statin, a lipid-lowering drug with well recognized cardiac protective effects commonly used in diabetic patients. Currently it remained difficult to say whether the positive association with some medications and the negative association with others could be due to the direct pharmacological effects of the medications or due to the indications or different disease severities related to their use. More studies are required to clarify the underlying mechanisms or explanations for the relationship with these baseline characteristics.

It is interesting that our previous studies also suggested that sitagliptin may increase the risk of acute pancreatitis [21], pancreatic cancer [22] and thyroid cancer [23] within one or two years of its initiation. The potential risk of these adverse outcomes associated with sitagliptin use have been previously reported and therefore a higher reporting rate might be expected due to clinical awareness and alertness, resulting in a detection bias. However, this could not be applied to the present study because the study period ended by the end of 2011, preceding the publication of most of the reports suggesting a potentially higher risk. There could also be a speculation on the reliability of the database on higher reporting rates of all types of cancer among patients who were initiated with sitagliptin. However, our unpublished analyses did not find such a significantly overall higher risk of other cancers among sitagliptin users, neither within one or two years after its initiation. Therefore, all of these pointed to a true link between sitagliptin use and the adverse outcomes including acute pancreatitis, pancreatic cancer, thyroid cancer and heart failure, especially during the initial period of its use. A similar observation of risk attenuation of these adverse outcomes after a longer duration of sitagliptin use [21–23] might be partly due to the depletion of susceptible cases.

There are some strengths in the study. First, we included all longitudinal data to cover the whole period since the availability of the database in 1996. Second, the large sample size representing the whole nation rendered the generalization of the findings to the whole population in a real world scenario. Third, the use of medical records reduced the bias related to self-reporting.

The limitations include a lack of laboratory data to support the diagnosis of heart failure. However, because the diagnosis of heart failure was made during hospitalization, the reimbursement for such hospitalization should be supported by laboratory data. Second, because no laboratory data were available to define the severity of heart failure, patients hospitalized with such a diagnosis might have represented those with more severe clinical symptoms. Third, the diabetes duration observed from the database may be underestimated because many patients with type 2 diabetes mellitus may remain undiagnosed for several years during the early phase of diabetes. Fourth, we did not have biochemical data such as blood levels of glucose and lipid profiles for evaluating their impacts. Fifth, this study could not evaluate the effects of other DPP4 inhibitors because only the sample size of sitagliptin users was large enough for evaluation.

In conclusions, the present study suggests a biphasic pattern in the association between sitagliptin use and heart failure hospitalization. Users with a cumulative duration < 1 year may show a significantly higher risk, but a significantly reduced risk can be seen in users with longer duration of exposure. This is the first study pointing out the potential protective effect of sitagliptin on heart failure after long-term use, suggesting that patients who have been using sitagliptin with a cumulative duration > 1 year should be kept on the medication to be benefited from its potentially protective effect on heart failure. Because spurious association could not be excluded among the short-term users, it remains to be clarified whether the significantly higher risk of heart failure among short-term users can be due to detection bias or residual confounding from the high prevalence of known risk factors of heart failure in the ever users of sitagliptin. The identification of some predictive and some protective baseline characteristics associated with sitagliptin-related heart failure is helpful for the physicians when they prescribe sitagliptin for blood glucose control to their patients.

## MATERIALS AND METHODS

An ethic review board of the National Health Research Institutes (NHRI) approved the study (approval number 99274). The identification information of each patient was scrambled, and written informed consent was not required according to local regulations.

The NHI was implemented since March 1995 and covers more than 99% of the Taiwanese population, with contracts covering 98% of the hospitals nationwide. The database keeps detailed records of the insurants' information of principal and secondary diagnostic codes, prescription orders, and claimed expenses from outpatient visits, emergency department visits, and hospital admission.

**Figure 1:**
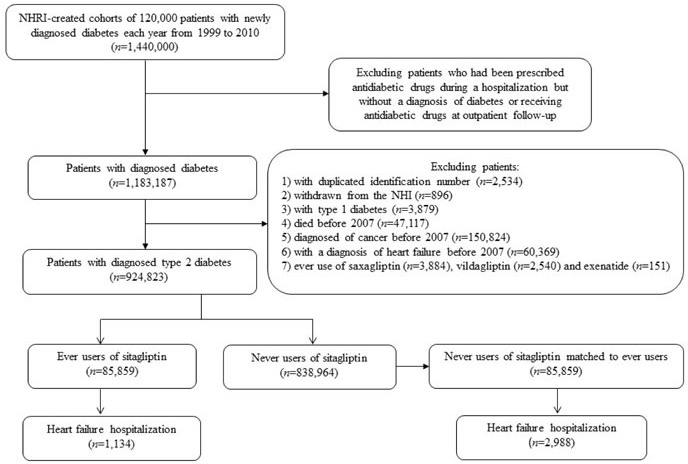
Flowchart showing the procedures followed in creating a cohort of 1:1 matched pair sample of sitagliptin ever and never users from the NHI for the study NHRI: National Health Research Institutes, NHI: National Health Insurance.

Figure 1 shows the flowchart for creating a cohort of 1:1 matched pair sample of sitagliptin ever and never users from the NHI. The NHRI created a cohort of 120,000 newly diagnosed diabetic patients in each calendar year for a 12-year period from 1999 to 2010 from the whole nation. The longitudinal reimbursement records of these patients from 1996 to 2011 can be provided for academic research. A patient should not have a diagnosis of diabetes in the previous years when he/she was randomly selected into the cohort for each specific year. The definition of diabetes was based on one of the following two criteria: 1) Diagnosis of diabetes during an admission to the hospital or having been prescribed with antidiabetic drugs during hospitalization; or 2) In an outpatient setting within one year, a patient has been diagnosed as having diabetes for two or more times, or diagnosed as having diabetes for one time plus prescribed with antidiabetic drugs for one time. As a result, a total of 1,440,000 patients with newly diagnosed diabetes were available within these 12 years.

In consideration that some patients might have been given insulin or oral antidiabetic drugs during an admission for some medical conditions but they might not be real cases of diabetes, patients who were recruited based on the criterion of having been prescribed with antidiabetic drugs during hospitalization but had not been followed at the outpatient clinics with a diagnosis of diabetes or had not received antidiabetic drugs at outpatient follow-up were first excluded. This resulted in a sample size of 1,183,187 patients. Patients who were alive on January 1, 2007 were recruited into the study by the following selection procedures. After exclusion of patients with duplicated identification number (*n* = 2,534), withdrawn from the NHI (*n* = 896), with a diagnosis of type 1 diabetes (*n* = 3,879), died before 2007 (*n* = 47,117), with a diagnosis of any cancer before 2007 (*n* = 150,824), with a diagnosis of heart failure before 2007 (*n* = 60,369), and ever use of saxagliptin (*n* = 3,884), vildagliptin (*n* = 2,540) and exenatide (*n* = 151, linagliptin and liraglutide were not available in Taiwan during the study period). As a result, a total of 924,823 patients were available. Among them, 85,859 patients had been newly prescribed with sitagliptin (ever users). To create a 1:1 matched pair sample of 85,859 patients who had not been treated with sitagliptin (never users), the methods described by Parsons based on 8 digits of PS derived from baseline characteristics by logistic regression were used [14]. These methods have also been used in our recently published papers [23, 24].

The *International Classification of Diseases, Ninth Revision, Clinical Modification* (ICD-9-CM) has been used during the study period and diabetes was coded 250.XX. Information of the first event of heart failure hospitalization (ICD-9-CM: 398.91, 402.11, 402.91, 404.11, 404.13, 404.91, 404.93 and 428) was linked from the hospitalization database.

The ICD-9-CM codes for the comorbidities were [25–29]: hypertension 401-405, chronic obstructive pulmonary disease (a surrogate for smoking) 490-496, stroke 430-438, nephropathy 580-589, ischemic heart disease 410-414, peripheral arterial disease 250.7, 785.4, 443.81 and 440-448, obesity 278, dyslipidemia 272.0-272.4, acute pancreatitis 577.0, alcohol-related diagnosis 291, 303, 535.3, 571.0-571.3, 980.0, and cancer 140-208. Medications included sulfonylurea, metformin, meglitinide, acarbose, pioglitazone, rosiglitazone, insulin, statin, fibrate, angiotensin-converting enzyme inhibitor, angiotensin receptor blocker, calcium channel blocker, aspirin, ticlopidine, clopidogrel, and dipyridamole.

The baseline characteristics of sitagliptin never users and ever users were compared by Student's t test for age and diabetes duration and by Chi-square test for others. The crude incidence density of heart failure hospitalization was calculated for sitagliptin ever users and never users and for the tertiles of cumulative duration of sitagliptin therapy (months). The numerator for the incidence was the number of patients with the first event of heart failure hospitalization during follow-up, and the denominator was the person-years of follow-up. Follow-up started on January 1, 2007 and ended on December 31, 2011, at the time of the first event of heart failure hospitalization, or at the date of the last reimbursement record. In the lack of information on the mortality or migration status of the patients, the last reimbursement record may serve as a surrogate because these patients should be withdrawn from the NHI in Taiwan. Standardized difference for each baseline characteristic was calculated according to the recommendation of Austin and Stuart and a value of > 10% may indicate meaningful imbalance with potential confounding [15].

The treatment effect was estimated by using PS-weighting with the IPTW approach incorporated into a Cox regression [16]. Hazard ratios were estimated forever users *versus* never users, and for each tertile of cumulative duration of sitagliptin therapy compared to never users as referent.

To identify the predictive and protective characteristics related to sitagliptin-associated heart failure, patients ever treated with sitagliptin were selected for a Cox regression model. In this model heart failure hospitalization was treated as the dependent variable and independent variables included all baseline characteristics.

Analyses were conducted using SAS statistical software, version 9.3 (SAS Institute, Cary, NC). *P* < 0.05 was considered statistically significant.
